# 

**Published:** 1899-05

**Authors:** 


### CONTENTS.

COMMUNICATIONS.

The Process of Refining and Manufacturing Gold Foil......... .. ..... 201
A Historical and Biographical Sketch of Geo. Watt, M.D., D.D.S. ... 209
Dr. George Watt ..................................................... 218

PROCEEDINGS.

Southern Branch, National Dental Association, Second Annual Meeting, New Orleans, La., February 9, 10, 11, and 13, 1899 ..... 223
"Operative Dentistry" ..................................... 223
"The Advantages offered in a Combination of Different Filling Materials ..................................... 224
Notes on Materia Medica, with Comments and Criticisms...... 225
The Treatment of Pulpless Teeth by Iodoform Fumes Under Pressure........................................ 227
Chemistry, Metallurgy and Anatomy.............................. 227
Defective Tooth Development.................................... 228
"Notes on Materia Medica, with Comments and Criticisms" 229
A Statement to American Dentists Practicing in Europe .............. 236
American Dental Society of Europe........................................ 239
The Dental Society of the State of New York.............................. 241

SELECTIONS.

Relations between Dentist and Patient .............................. 241
An Unpleasant Experience with Cocaine.................................. 243
The Influence of the Quality of Drinking Water in the Structure of the Teeth.................................................. 245
On Recent Methods of Treating Diseased Pulp ........................ 246
Nitro-Hydrochloric Acid in Dentistry.................................... 247
Abnormality of Hair Growth with Total Absence of Teeth ........... 248
Stuttering .......................................................... 248
The Best Disinfectant............................................... 248

NOTICES.

International Dental Congress, Paris, 1900............................. 249
The Annual Meeting of the National Association Dental Faculties... 249

EDITORIAL.

Commencements.......................................................... 250

